# Folding Pathways of a Knotted Protein with a Realistic Atomistic Force Field

**DOI:** 10.1371/journal.pcbi.1003002

**Published:** 2013-03-21

**Authors:** Silvio a Beccara, Tatjana Škrbić, Roberto Covino, Cristian Micheletti, Pietro Faccioli

**Affiliations:** 1 LISC, Bruno Kessler Foundation, Trento, Italy; 2 ECT*, Bruno Kessler Foundation, Trento, Italy; 3 Physics Department, University of Trento, Trento, Italy; 4 INFN, Gruppo Collegato di Trento, Trento, Italy; 5 SISSA and CNR-IOM Democritos, Trieste, Italy; Iowa State University, United States of America

## Abstract

We report on atomistic simulation of the folding of a natively-knotted protein, MJ0366, based on a realistic force field. To the best of our knowledge this is the first reported effort where a realistic force field is used to investigate the folding pathways of a protein with complex native topology. By using the dominant-reaction pathway scheme we collected about 30 successful folding trajectories for the 82-amino acid long trefoil-knotted protein. Despite the dissimilarity of their initial unfolded configuration, these trajectories reach the natively-knotted state through a remarkably similar succession of steps. In particular it is found that knotting occurs essentially through a threading mechanism, involving the passage of the C-terminal through an open region created by the formation of the native 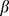-sheet at an earlier stage. The dominance of the knotting by threading mechanism is not observed in MJ0366 folding simulations using simplified, native-centric models. This points to a previously underappreciated role of concerted amino acid interactions, including non-native ones, in aiding the appropriate order of contact formation to achieve knotting.

## Author Summary

It has been recently observed that the native structure of proteins can contain knots. These are formed during the folding process and are tightened in a specific (i.e. native) location, along the poly-peptide chain. The existence of knots hence implies a high degree coordination of local and global conformational changes, during the folding reaction. In this work we investigate how the knot is formed and what are the dynamical mechanisms which drive the self-entanglement process. To this end, we report on the first atomistically detailed numerical simulation of the folding of a knotted protein, based on a realistic description of the inter-atomic forces. These simulations show that the knot is formed by following a specific sequence of contacts. The comparison of the findings with those based on simplified folding models suggest that the productive succession of contacts is aided by a concerted interplay of amino acid interactions, arguably including non-native ones.

## Introduction

Natively-knotted proteins are increasingly studied as a new paradigm of “multiscale” folding coordination, which leads to establishing the native knot in the native position starting from the unknotted newly-translated state [1]–[4]. Intuitively, the pathways associated to this process appear so improbable and prone to misfolding that it was long held that naturally occurring proteins would be protected against the occurrence of knots. This *a priori* expectation, which has a sound statistical basis [5], [6], was so strong radicated that only several years after the publication of the human carbonic anhydrase II structure [7] it was realized that it actually accommodated a knot [8]. Since then, hundreds of instances of naturally-occurring knotted proteins have been found and they now account for about 2% of the protein data bank (PDB) entries [6].

The salient aspects of the folding phenomenology of several knotted proteins have been recently probed by various experiments (for recent reviews see refs. [1]–[4]). These studies have demonstrated that newly translated, unknotted proteins, can fold into the native knotted structure without the assistance of chaperones [9], [10], though the latter can significantly speed up the process [10]. The details of the concerted backbone movements that lead to the self-tying of the protein in the native knot remain, however, beyond reach of current experimental techniques. In this regard, numerical investigations can aptly complement experimental ones, by providing valuable insight into the repertoire of viable modes of knot formation, the stage at which the knot is formed, and the possible role of non-native interactions [11]–[14].

To ease the major computational burden imposed by simulating the slow process of spontaneous folding/knotting of these molecules, the above-mentioned studies were performed using 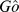-type native-centric force fields, in either coarse-grained (CG) or atomistic protein representations. The latter approach allowed for establishing the noteworthy result that by promoting native interactions alone it is possible to fold a natively-knotted protein [11], [12]. Non-native interactions have, however, been argued to be important for enhancing the efficiency of the process, by significantly increasing the accessibility of knotted configurations in the early folding stages [13], [14].

A natural test case for numerical studies of spontaneous knotting in polypeptide chains is represented by protein MJ0366, which is the shortest known knotted protein. The folding process of this 82-amino acid long protein appears to be governed by such a delicate interplay of amino acid stereochemical interactions that folding simulations employing different levels of spatial resolution have been shown to yield different knotting mechanisms. In particular, the seminal study of Noel and co-workers [12], where the folding of MJ0366 was characterized using pure native-centric force-fields, has shown that in coarse-grained folding simulations, the knot could form at either terminus, while only the C-terminal is involved in knotting when the full atomistic detail is used.

The observed sensitivity of the MJ0366 folding process on structural details poses a further fundamental question: to what extent is the knotting mechanism sensitive to details of the force field used in folding simulations?. Towards this goal, we here analyze an ensemble of about 30 successful atomistic folding trajectories for protein MJ0366, obtained by using a realistic force field, namely AMBER99ffSB [15] with implicit solvent.

To the best of our knowledge this represent the first instance where a realistic force-field is employed to follow the folding of initially unfolded, and unknotted conformations into a knotted native state.

To collect this sizeable number of productive trajectories in an affordable amount of computational time, we have used an advanced simulation technique known as the “dominant reaction pathway” (DRP) scheme. In other protein contexts, this method was shown to yield results consistent with standard extensive MD folding simulations, performed with the same atomistic force field [16].

We find that self-knotting of MJ0366 typically occurs at a late folding stage, when about 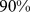 of the native contacts are established and almost invariably involves a single dominant knotting mechanism. The latter consisting of the threading of the C-terminus through an open region created by an already formed 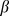-sheet. Based on various model calculations it is argued that the observed difference in knotting modes is strongly influenced by non-native interactions.

## Results/Discussion

**Figure 1:**
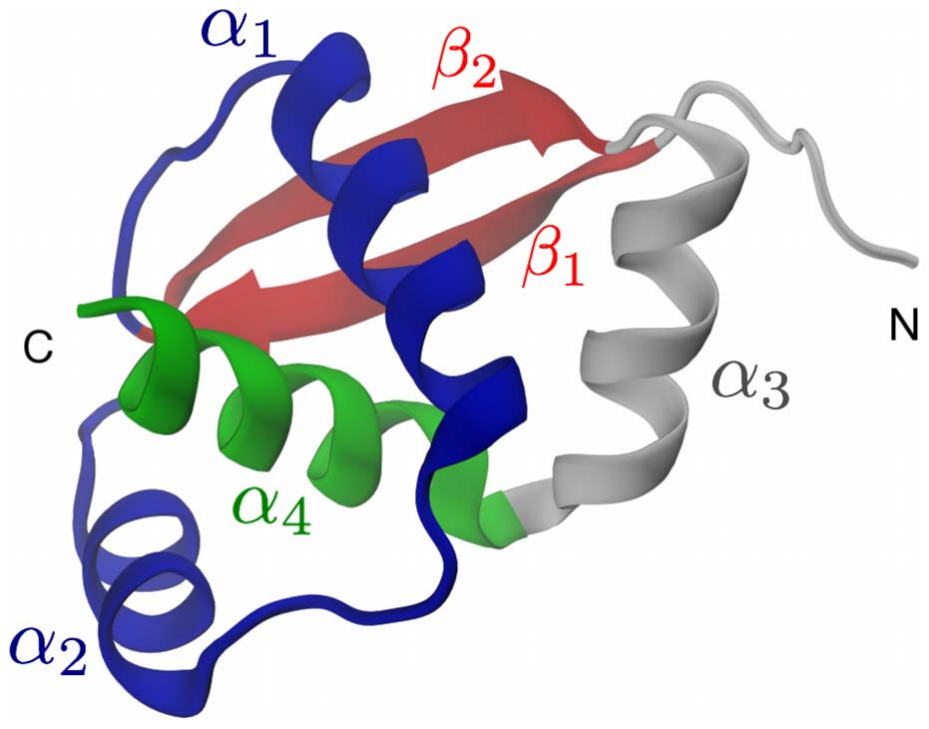
Crystal structure of protein MJ0366, PDB code: 2EFV. This and other images were rendered with VMD [38].

The monomeric unit of the natively-knotted MJ0366 protein consists of 82 amino acids and comprises four 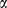-helices and one 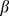-sheet resulting from the pairing of two antiparallel strands with a large sequence separation (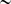
30 amino acids), see Fig. 1. The C-terminal helix, 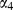, protrudes through a loop formed by the other two 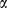-helices giving rise to a rather shallow trefoil-knot.

We report on the characterization of the folding process of MJ0366 by means of advanced molecular dynamics techniques based on the AMBER99ffSB atomistic force field [15] with implicit solvent. The numerical strategy was articulated over several steps.

Specifically, we first generated an ensemble of 100 denatured configurations for protein MJ0366 by unfolding the crystal structure using 100 ps of atomistic molecular dynamics (MD) simulations at high temperature (1600 K) followed by 100 ps of thermalization at 300 K.

Next, the folding and knotting dynamics of MJ0366 was studied by carrying out 40–50 folding attempts for each of the 100 denatured configurations, for a total of about 4000 attempted folding trajectories. Simulating so many folding trajectories from an initially unfolded state is presently beyond reach of standard MD simulations even when run on dedicated supercomputers [17]. To overcome these difficulties we resorted to the recent development of the DRP approach proposed in ref. [16]. This combines a ratchet-and-pawl molecular dynamics algorithm [18], [19] (rMD) with a statistical analysis based on scoring *a posteriori* the relative likelihood of each computed folding pathways [20]–[23]. This method is described in detail in the next section, and has been recently used to investigate the folding of the WW domain FIP35 [16] yielding a very consistent folding mechanism with ms-long MD simulations in explicit solvent [17].

The strength of the rMD scheme is that it allows for efficiently generating an ensemble of trial folding pathways from a given initial denatured state to the known native state, while keeping at a minimum the external work applied to drive the system. In fact, the system dynamics evolves in a completely unbiased way whenever it leads to a higher similarity with the native state, i.e. a larger number of formed native contacts. Conversely, a time-dependent external force is introduced to discourage, though not completely prevent, a decrease of the native similarity. The biased rMD evolution promotes only the overall geometrical similarity with the native state and does not reward specific concerted backbone movements that could lead to knotting. As a matter of fact, knot formation was observed only for a small fraction of the thousands of attempted rMD trajectories, namely 66 of them, covering 31 distinct initially-unfolded states. In all cases, the knotting event corresponded to the formation of the native trefoil knot, thus indicating that incorrect knot formation is not a major source of kinetic trapping for MJ0366.

In the DRP approach, only one productive pathway per initial condition was retained, namely the one with the highest statistical weight. This weight, corresponds to the probability that each trial trajectory is generated by an overdamped Langevin dynamics. Notice that, because the weights are calculated with reference to an *unbiased* stochastic dynamics, the DRP selection criterion lessens *a posteriori* the rMD steering effects.

### Trajectories analysis

The selected 31 trajectories were analyzed by monitoring the evolution of several geometrical and topological parameters during the folding process.

**Figure 2:**
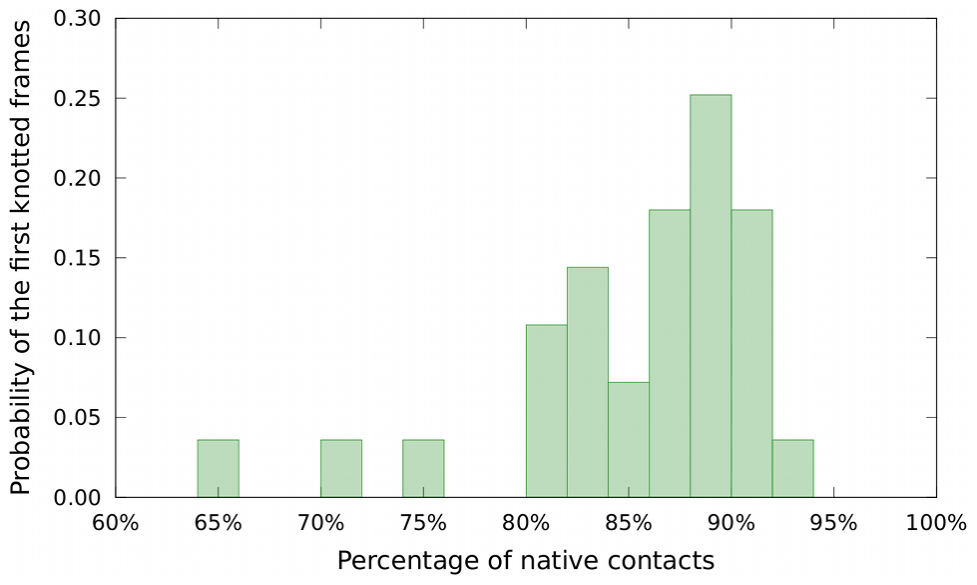
Distribution of the percentage of formed native contacts at the time of the first knotting event for the 26 DRP trajectories of MJ0366.

As a first step we identified the folding stage at which the backbone self-ties into knot. Accordingly, for each trajectory we calculated the percentage of native contacts (overlap) that are formed when the first knotting event occurs. The distribution of these overlaps for the considered trajectories is shown in Fig. 2. The distribution is peaked at about 90% overlap. This indicates that the knot is typically formed at a rather late stage of the folding process.

Next, to characterize the diversity of the folding pathways and the implications for the knotting mechanism, we computed the average “path similarity parameter”, 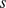. As explained in the Materials and Methods section, this quantity measures the consistency of the temporal succession in which the native contacts are formed in two given pathways. The 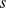 parameter takes on values ranging from 0, for no similarity, to 1 when all native contacts form with exactly the same succession in the two trajectories. We emphasize that 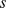 depends only on the time order of native contact formation events (and not their exact timing).

**Figure 3:**
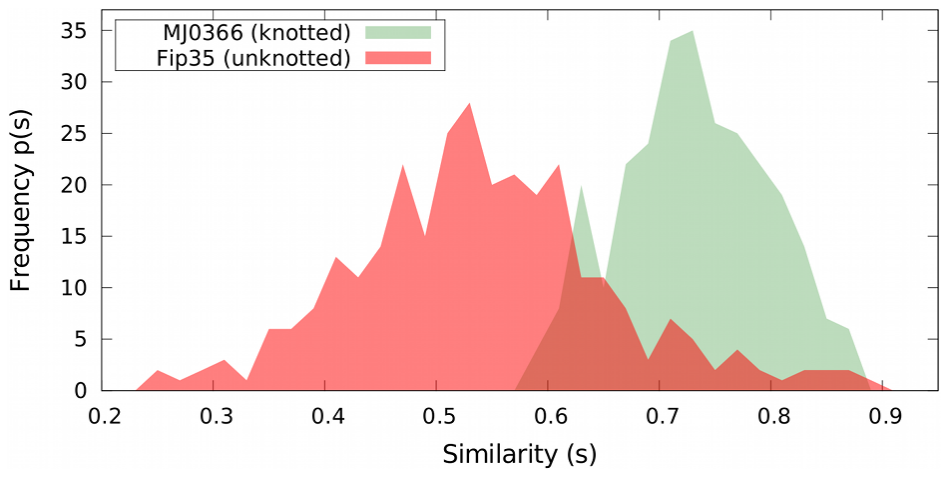
Distributions of the path similarity parameter 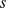, see Eq. 7 for DRP trajectories. The green distribution pertains to the 26 DRP trajectories of the knotted protein MJ0366. For comparative purposes, the red curve shows the 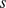 distribution of DRP trajectories of the uknotted WW domain FIP35 [16].

To have a robust indication of the degree of heterogeneity of the selected trajectories, we computed the distribution of 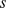 over all possible pairs of trajectories, see Fig. 3. As a term of reference, the same Figure shows the 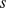 distribution computed over previously-studied folding trajectories of the unknotted WW domain FIP35 [16]. It is seen that the distribution of MJ0366 is narrower and shifted towards significantly higher values of 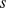 than for the unknotted protein. Indeed the former has a peak at 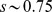 while the latter has it at 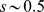. This relatively low value of 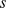 and the distribution broadness is typical of folding processes that proceed by multiple pathways [16], [24], as FIP35 is known to do. The different characteristics of the 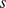 distribution for MJ0366 therefore strongly suggest the existence of one dominant folding pathway for MJ0366.

We accordingly sought to analyze in detail the folding process to verify that knotting occurs via one dominant mechanism and characterize it.

In this regard a valuable term of reference is given by the earlier study of Noel *et al.* [12] where the folding thermodynamics of MJ0366 was systematically characterised with both atomistic and coarse-grained native-centric models. When the atomistic model was employed, it was seen that knotting preferentially occurred via slipknotting. Specifically, in most of the productive trajectories obtained at the folding temperature of the structure-based model, the C-terminal attained a hairpin-bent conformation and established the knot by threading the open region involving residues 17–54. The slipknotting mechanism was found to occur more frequently than that of other knotting modes, such as the threading of the open region by a non-bent C-terminus, or knot formation at the N terminus. Interestingly, the coarse-grained native-centric model was more prone to unproductive kinetic traps and displayed significant heterogeneity for knotting mechanisms too. These aspects indicated that the realistic treatment of protein structural detailed significantly helped reduce the impact of unproductive routes in the folding process [12].

Here, by addressing the same protein folding process with a realistic, non native-centric force-field, it is possible to examine to what extent various aspects of the knotting process are sensitive to the treatment of inter-atomic interactions.

As a first step of the analysis, we profiled the folding trajectories along two relevant order parameters: the root mean square distance (RMSD) to the native structure and the RMSD to the native 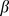-sheet. The first collective variable monitors the overall progress towards the native geometry. The second one, instead, carries information about one of the expected entropic bottlenecks of the folding process, namely the formation of the native antiparallel 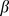-sheet which involves amino acid pairs with a sequence separation as large as 38.

Since in the native MJ0366 structure the C-terminal helix protrudes through the region intervening between the two paired 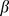-strands, monitoring the formation of the 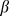-sheet is relevant to understand whether the sheet is formed before or after the knot.

**Figure 4:**
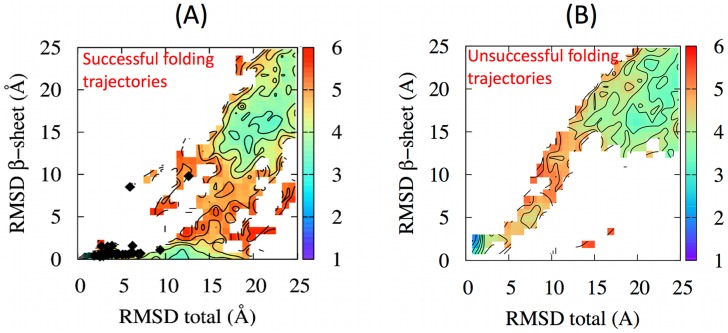
Atomistic DRP folding pathways, projected on the plane selected by the total RMSD to native and by the RMSD to native of the 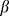-sheet. Panels (A) and (B) refer respectively to successful and unsuccessful folding trajectories. The diamonds in panel (A) mark the collective coordinates at the time of knot formation. The scale on the left corresponds to the logarithm of the number of times a given spot is visited by the DRP trajectories, in analogy with free-energy landscape plots.

The results shown in the left panel of Fig. 4 indicate that the 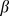-sheet is fully formed rather early, when the total RMSD to native of the chain is about 15 Å. At this stage the fraction of formed native contacts is about 40–50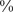. The self-tying of the molecule into a trefoil knot typically occurs after the formation of the 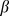-sheet. This is evident from the placement of the diamond symbols in Fig. 4 which mark the first occurrence of knots for each of the 31 trajectories. It is seen that all first-knotting events occur when the 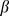-sheet is fully formed, with only two exceptions that will be discussed later.

**Figure 5:**
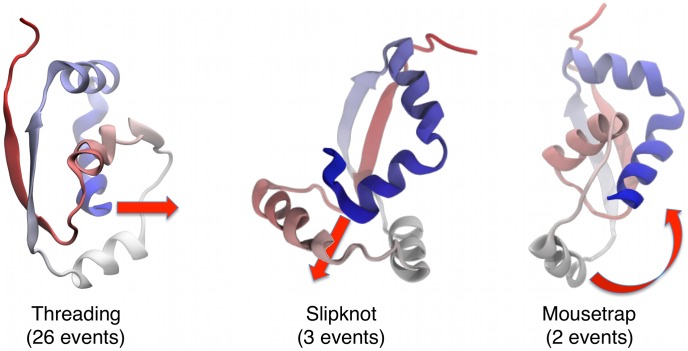
The three different types of knotting mechanisms observed in our atomistic DRP simulations.

The detailed inspection of the trajectories indicates that the knotting process almost invariably occurs through the so-called “threading” mechanism, where the 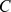-terminal 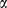-helix (residues 74–87) directly enters, without bending, the open region between amino acids 17–54 involving helices 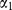 and 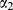 and the intervening loop, see the sketch in the left panel of Fig. 5. In this case, the threaded region and the 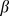-sheet (respectively shown in blue and red in Fig. 1) establish a tertiary contact before the terminal helix penetrates into the open region in between the helices 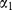 and 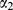 (see left panel in Fig. 5). This mechanism accounts for as many as 26 of the 31 rMD trajectories.

In three other cases, the folding was found to occur through the so-called “slipknot” mechanism [12] (i.e. where the open region is entered by the backward-bent C-terminus). In all three instances the 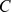 terminus entered the loose 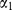
–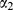 region producing a shallow slipknotted trefoil, as shown in the central panel of Fig. 5.

Finally, in two further cases we observed another knotting mechanism which involves a concerted backbone movement that had not been previously reported for MJ0366. Specifically, in two trajectories when the 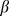-sheet and the terminal 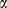-helix are already formed and juxtaposed in an unknotted configuration the loop performs a “mousetrap-like” movement establishing the native knotted topology. This movement, which bears some analogies with the suggested knotting mechanism for an unrelated protein with a non-trefoil topology [25], is schematically represented in the right panel Fig. 5. The mousetrap knotting events correspond to the two outlying diamonds reported in Fig. 4, with collective coordinates (6 Å, 8 Å) and (12 Å, 10 Å).

Videos obtained from the atomistic DRP trajectories which illustrate the three observed knotting mechanisms are included in the on-line SI.

**Figure 6:**
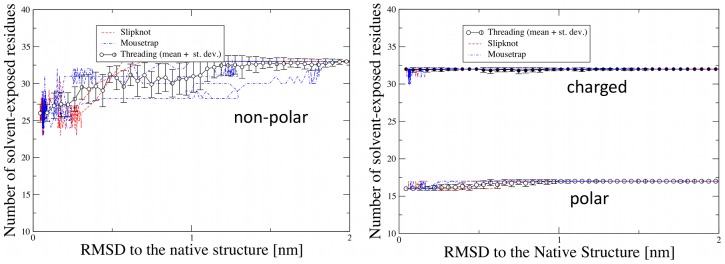
Exposure to the solvent of polar, non-polar and charged residues along the folding trajectories pertaining to three different knotting mechanisms, plotted as a function of the RMSD to the native structure. The number of amino acids exposed to the solvent was computed using the VMD utility [38]. The dashed and dot-dashed lines represent folding events with mousetrap and slipknotting mechanism, respectively. The points are the average over the 26 DRP trajectories with a threading knotting mechanism, and the error bars denote the corresponding standard deviation. Left panel: evolution of non-polar residues; Right panel: evolution of polar and charged residues.

It is important to notice that the trajectories associated to the various knotting modes do not present significant quantitative differences regarding the overall solvent accessibility of polar and non-polar residues during the folding process. This point is illustrated in Fig. 6 where the number of exposed hydrophobic and hydrophilic residues are profiled versus the RMSD to the native state. The consistency of the various profiles provides a quantitative basis for expecting that the relative weight of the knotting mechanisms should not depend critically on the specific model adopted to describe the solvent-induced interactions.

### Order of contact formation and knotting

To understand how the interplay of amino acid interaction captured by the realistic force field favours knotting by threading, we have carried out a comparative analysis of the reaction mechanism in successful and unsuccessful folding trajectories. Specifically, the productive, successful set consisted of the 26 trajectories displaying the dominant (threading) knotting mechanism. The non-productive one included an equal number of trajectories that reached an *unknotted* configuration and nevertheless had a good native similarity (namely an RMSD to the crystal structure less than 5 Å).

The projection of the unsuccessful trajectories along the two collective coordinates considered before is shown in Fig. 4B. The qualitative difference with respect to the analogous plot for the successful ones (panel A) is striking. In particular, it is seen that in successful trajectories the formation of the sheet involving strands 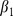 and 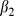 occurs rather early on and prior to the establishment of the overall tertiary organization of MJ0366. In fact, the total RMSD to native decreases appreciably only after the 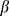-sheet is established. By converse, for unsuccessful trajectories, this hierarchy of contacts formation is not observed, and the 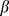-sheet formation proceeds in parallel with the acquiring of the overall native structure. One therefore concludes that the early formation of the 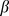-sheet provides the most appropriate conditions for knotting by leaving the region delimited by the 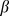 sheet accessible to threading events.

**Figure 7:**
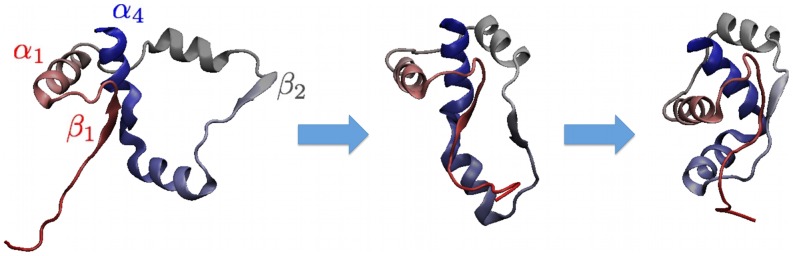
Typical example of unsuccessful trajectory. The late formation of the 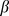-sheet traps the 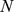 terminus on the “wrong” side of the 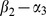 loop and prevents attaining the (native) knotted topology.

This conclusion is supported by the detailed inspection of the unsuccessful trajectories, which are exemplified in the sequence of snapshots shown in Fig. 7. As it is visible in this figure, the C-terminal helix threads the correct region between strands 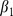 and 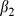 prior to the formation of the 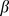-sheet. When the latter is finally establishes, the 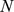-terminal segment remains trapped on the wrong side of the loop bridging 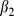 and 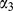 and, for steric reasons cannot go past it and attain the native knotted topology.

The relevance of this mechanism for misfolding is highlighted by the fact that all unsuccessful trajectories displayed a late formation of the 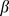-sheet. We emphasize again that, according to our simulations, the correct knotting of the chain is not promoted by the formation specific contacts which fail to form in misfolding events. Rather, for the chain to acquire the native topology, it is essential that the native contacts form in the correct order.

### Further insight from coarse-grained models

The fact that the observed dominant knotting mode differs from the one reported previously using pure native-centric force fields suggests that non-native interactions could be relevant for favouring the correct succession of contacts leading to self-knotting (or avoiding unproductive ones). This possibility is particularly interesting in connection with the ongoing discussion about the role that non-native interactions can have in aiding the knotting process even during the early folding [13], [14].

To investigate this aspect we generated several folding trajectories for MJ0366 using simplified models where the effect of non-native interactions could be easily turned on or off. Specifically, we considered two different coarse-grained models: one with only native-centric interactions and the other additionally incorporating non-native interactions. The latter included quasi-chemical and screened electrostatic pairwise interactions, as in the recent study of the early folding stages of a trefoil-knotted carbamoyltransferases [14].

The folding process presents major differences in the two models. First, they differ significantly in terms of knotting probability. Specifically, for each model we considered an extensive set of 10,000 uncorrelated configurations, equilibrated at the nominal temperature of 300 K. In the native-only case, 12% of the sampled configurations were knotted, while this number had a sixfold increased, to 75%, in presence of non-native interactions. This result aptly complements the atomistic DRP simulations, for highlighting the role of non-native interactions in aiding the formation of the native knotted topology of MJ0366.

Secondly, productive trajectories follow different dominant mechanisms in the two models. In fact, when the pure native-centric model is used, 8 out of the 10 trajectories involved the slipknotting mechanism, while the threading one was observed in all trajectories (10 out of 10) with the additional non-native interactions. The latter result, which is in full accord with the atomistic DRP simulations, reinforces the concept that non-native interaction can promote the correct order of contact formation required for self-knotting.

**Figure 8:**
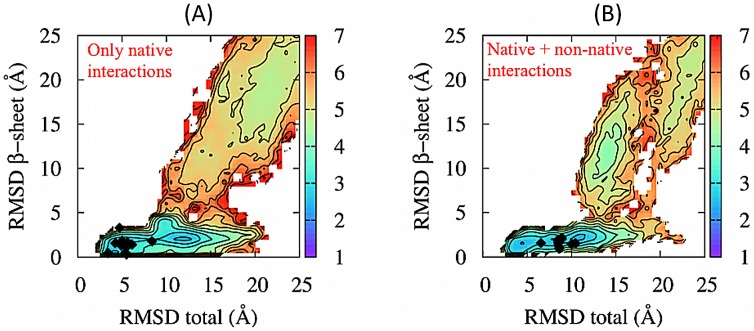
Folding pathways obtained from coarse-grained Monte Carlo simulations with local crankshaft moves which mimic the chain dynamics, projected on the plane selected by the total RMSD to native and by the RMSD to native of the 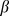-sheet. Panel (A) refers to the model with only native interactions, while panel (B) refers to the model with both native and non-native interactions. The diamonds denote the values of the collective coordinates at the time of knot formation. The scale on the left is the logarithm of the number of times the point is visited by folding trajectories, in analogy with free-energy landscape plots.

This point is further supported by the inspection of the density plots in Fig. 8. In fact, non-native interactions are more clearly associated to the early formation of the 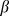-sheet than for the native-only case. Furthermore, the path outlined in panel B bears more analogies than the one in panel a with the density plot of Fig. 4A, which captured the successful folding events obtained from atomistic DRP simulations. Indeed, in the simplified model, the early formation of the 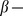
sheet is promoted by the fact that the non-native quasi-chemical interaction generates an overall attractive interaction between the residues in 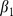 and those in 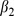. Consistently with the misfolding events discussed previously, one can therefore argue that the weaker drive of the native-centric model to form early on the 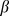-sheet, is also responsible for its lower knotting propensity.

Based on these results, we can argue that mutations in the 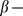
sheet regions with residues characterized by a weaker effective attraction, would delay the formation of the 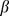-hairpin in the folding process and would make the chain more prone to reach the unknotted mis-folded state. This prediction may be verified experimentally.

### Concluding remarks

In conclusion, the DRP simulations presented here provided the first systematic attempt to characterize the folding process of a natively-knotted protein, MJ0366, using a realistic atomistic force field. MJ0366 knotting is observed to occur via threading at the C-terminal. The comparison of productive and unproductive trajectories (which respectively end up in natively-knotted and unknotted states) further indicates that knotting is aided by the early formation of the native 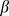-sheet. By comparing the MJ0366 knotting propensity and mechanisms in simplified folding models it is argued that non-native interactions are important for aiding knotting by promoting the correct order of contact formation.

While there is no *a priori* reason to expect that non-native interactions are crucial for guiding the folding process of knotted proteins in general, it is interesting to notice that their important role has been previously suggested for another trefoil-knotted protein carbamoyltransferases [13], [14]. In our view, it would be most interesting to further examine this effect, in future studies on MJ0366 or other proteins either through experiments (e.g. involving mutagenesis) or with more extensive simulations, possibly involving explicit solvent treatment or unbiased dynamics.

## Materials and Methods

### The rMD and DRP algorithms

In order to generate an ensemble of trial trajectories connecting a given initial configuration to the native state we used the following variant of the rMD algorithm. At each integration step, we evaluated a collective coordinate (CC) which measures the distance of between the instantaneous contact map and the native contact map:
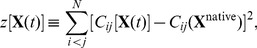
(1)with 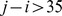 with a distance cutoff of 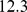 Å. In this equation, 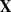 is the 3N-dimensional vector in configuration space, and 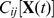 and 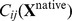 are the instantaneous and native contact map, respectively. The entries of the contact map C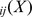 are chosen to interpolate smoothly between 0 and 1, depending on the relative distance of the atoms 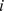 and 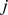:
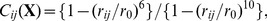
(2)where r_0_ = 7.5 Å is a fixed reference distance.

In the rMD algorithm, no bias is applied to the chain when it spontaneously diffuses towards the bottom of the folding funnel, i.e. any time the value of the CC at time 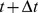 is smaller than the minimum value so far. On the other hand, fluctuations which would drive the contact map further from the native one (hence increasing 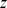) are hindered by introducing a biasing force, defined by the time-dependent potential
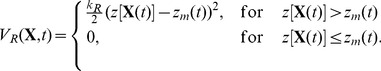
(3)In these equations, 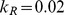 kcal/mol is the so-called ratchet constant and 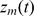 is the minimum value assumed by the collective variable 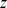 along the rMD trajectory, up to time 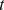.

In the original formulation of the rMD algorithm [19], the variable 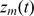 is updated only when the system visits a configuration with 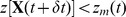. With this choice, 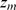 monotonically decreases during the course of the simulation. In this work, we choose to significantly weaken the effect of the bias by allowing the system to backtrack along the direction defined by the CC. This is done by occasionally updating 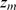 also when it increases, according to a Metropolis accept/reject criterium. Namely, 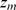 is updated to 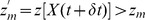 if 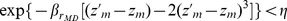, where 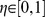 is a random number sampled from a uniform distribution and 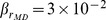 is an artificial “inverse thermal energy”. This modification of the original rMD algorithm is required to escape from kinetic traps. Without it the folding efficiency to the correct topologically non-trivial native state is strongly suppressed. Each trial trajectory consisted of 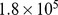 steps of rMD with a nominal integration time step of 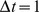 fs.

The DRP algorithm is used to identify the most probable path in each set of trial rMD trajectories sharing the same boundary conditions. This is done by evaluating the relative probability for each path 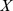 to be realized in the unbiased over-damped Langevin dynamics:
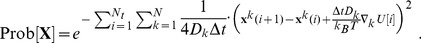
(4)In this equation, the index 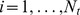 runs over the different time-step in the trajectory, the index 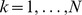 runs over the atoms in the protein, 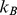 is the Boltzmann's constant and 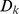 is the diffusion coefficient of the 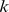-th atom.

### Atomistic force field

In both rMD and standard high-temperature MD simulations we used the AMBER ff99SB force field [15] in implicit solvent, within the Generalized Born formalism implemented in GROMACS 4.5.2 [26]. In such an approach, the Born radii are calculated according to the Onufriev-Bashford-Case algorithm [27]. The hydrophobic tendency of non-polar residues is taken into account through an interaction term proportional to the solvent-accessible-surface-area (SASA). The solvent-exposed surface of the different atoms is calculated from the Born-radii, according to the approximation developed by Schaefer, Bartels and Karplus in [28].

### Alternative simplified force fields

The CG folding simulations were based on the model developed in Ref.s [29], [30]. In this approach, amino acids are represented by spherical beads centered at the 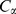 positions. The non-bonded part of the potential energy contains both native and non-native interactions. The former are the same used in the G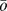-type model of Ref. [31], while the latter consist of a quasi-chemical potential, which accounts for the statistical propensity of different amino-acids to form contact and of a Debye-screened electrostatic term. A detailed description of the force field of this model can be found in Ref. [14]. In our previous work, we have shown that such non-native interactions are able to strongly promote the knot formation in natively knotted polypeptides [14]. Folding simulations for protein MJ0366 in this CG model were performed using a MC algorithm described in detail in Ref. [14]. This type of crankshaft-based MC algorithm is commonly employed in polymer physics [32] to study dynamic properties, since it is was shown that they mimic the intrinsic dynamics of a polymer in solution [33] at a much lower computational cost than standard MD simulations [34]. The folding dynamics of CG model with native and non-native interactions was simulated by generating 200 MC trajectories, while the dynamics of the model with only native interactions was investigated by generating 500 MC trajectories. For both CG models, trajectories consist of 1.5
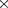
10^8^ attempted MC moves, corresponding to 1.5
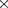
10^4^ saved frames. MC moves that we have employed were the local crank-shaft and Cartesian moves, whose boldness was chosen such that the acceptance rate was nearly constant and approximately equal to 50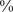. In both cases, we have collected a total of 10 folding transitions, leading to native configurations with the correct knotted topology. In order to compute the frequency of knotted configurations at thermal equilibrium we performed MC simulations which combine local moves and global pivot moves.

### Knot detection

The conformations visited during the MC dynamics were analysed to establish their global and local knotted state. The global topological state was established and assigned by computing the Alexander determinants after suitable closure of the whole protein chain into a ring. For each configuration, this entailed 100 alternative closures where each terminus is prolonged far out of the protein along a stochastically chosen direction, and the end of the prolonged segments are closed by an arc “at infinity” (i.e. not intersecting the protein). As in ref. [14], to avoid considering back-turning closures, stochastic exit directions are picked uniformly among those which form an angle of more than 90° with the oriented segment going from each terminus to the C*_α_* at a sequence distance of 10. If the majority of the 100 stochastic closures return non-trivial Alexander determinants, then the whole conformation can be considered as globally knotted. Because protein knotting can occur through slipknot formation [35], the global topology investigation was complemented by a local one. In fact, a slipknot can be detecting by identifying a non-trivially knotted portion of a chain that has a different global topology, in our case the unknotted one. To this purpose, we repeated the above-mentioned statistical closure scheme for all possible subportions of length 20, 30, 40, … of the protein chain so to identify the smallest knotted, or pseudo-knotted, chain portion [36], [37].

### Path similarity

To quantitatively measure the folding pathways diversity we implemented the analysis described in Camilloni et al. [19], that will be shortly summarized in the following. A folding mechanism is here considered to be a specific sequence of native contacts formation. Hence, for each path we measured the time of formation of each native contact, as the frame of the trajectory where the contact is first formed. Given 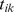 as the time of formation of the 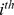 native contact in the 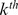 trajectory, we computed for each path 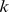 the matrix 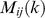 defined as:
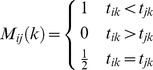
(5)containing all the information regarding the folding mechanism as defined above. For each pair of pathways 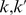 it is possible to compute the similarity 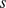 defined as
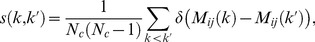
(6)

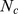
 being the total number of native contacts. The similarity ranges from 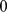 for completely different mechanisms, to 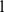 for completely identical mechanisms. Finally, we consider the distribution
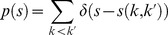
(7)of the similarity parameter, evaluated from all pairs of the folding pathways.
